# Developing a safety and health practices in building model of physical environment, facility management, and worker perception: Structural equation modeling approach

**DOI:** 10.1016/j.heliyon.2024.e40396

**Published:** 2024-11-14

**Authors:** Mohammad Lui Juhari, Kadir Arifin, Kadaruddin Aiyub, Zitty Sarah Ismail

**Affiliations:** a Centre for Research in Development, Social and Environment (SEEDS), Faculty of Social Sciences and Humanities, National University of Malaysia, 43600 UKM Bangi, Selangor, Malaysia; b Universiti Teknologi MARA, Department of Chemistry and Environment, Faculty of Applied Sciences, Shah Alam, Selangor, Malaysia

**Keywords:** Facility management, Physical environment, Perception of office workers, SEM, Occupational safety and health

## Abstract

Facility management is essential in modern office settings, as it helps to ensure the safety and health of employees. Issues related to facility management weaknesses, such as facility damage, structural issues, ventilation problems, and more, are subjects of ongoing concern. Therefore, safety and health practices are important aspects of facility management to ensure that the office is always safe and healthy for employees. *Objectives*: This research aims to address these concerns by developing a comprehensive model for safety and health practices in building, focusing on the physical environment, facility management, and worker perception. *Methods*: Data was gathered through random survey questionnaires distributed to government offices within the federal territory of Putrajaya, Malaysia. The survey covered 156 measurement items across 18 parameters, encompassing three dimensions: facility management, the physical environment, and office workers' perceptions. A total of 562 valid responses were collected. The data was analyzed using Structural Equation Modeling (SEM), which integrates path analysis and confirmatory factor analysis (CFA), through the IBM SPSS-AMOS software. *Results*: The results of this research indicate that the analysis of the model's goodness of fit proves that all variables within the model, whether independent or dependent are fitted and can be adapted. Consequently, the safety and health practices in building model was successfully developed. *Conclusion*: This model offers a valuable tool for facility management organizations, enabling them to systematically assess and maintain safe and healthy workplace conditions. It signifies a significant step forward in ensuring employee well-being in office buildings.

## 1 Introduction

In a world experiencing rapid urbanization, the effective management of facilities within urban environments has become a focal point of concern. The safety and health of employees are intricately linked to the oversight of these spaces, presenting a global challenge. Across the world, organizations grapple with the complex task of maintaining and improving their workplace structures while ensuring the well-being of their employees [[1], [2], [3]].

This global context has set the stage for a comprehensive exploration of workplace facility management, particularly with an emphasis on physical environmental factors such as indoor air quality, lighting, noise levels, heat, and cleanliness [4,5]. Workplaces worldwide encounter common challenges when managing their facilities, including the significant issue of ageing infrastructure. Many workplaces are home to older buildings that require maintenance and may pose safety risks. The process of refurbishing and rejuvenating these structures, all the while considering indoor air quality, lighting, noise levels, and cleanliness without causing disruptions, presents a considerable challenge. Moreover, resource constraints are another obstacle faced by facility management, with budget limitations and scarcity of resources often hindering the maintenance of clean air, suitable lighting, acceptable noise levels, and overall cleanliness [6]. In addition, the effective management of workplace facilities necessitates a skilled and well-trained workforce, particularly in matters related to indoor air quality, lighting, noise, and cleanliness [[7], [8], [9]]. Having qualified personnel and ongoing skills development is vital, especially considering evolving safety and health legislation at local, national, and international levels [[6], [7], [8], [9], [10]].

However, according to Hauashdh et al. the effectiveness of workplace facility management goes beyond physical attributes [11]. It is equally reliant on the perceptions and experiences of the individuals who utilize these spaces daily. Office workers' perceptions play a critical role in influencing their feelings about the workplace environment and its impact on their well-being. These perceptions encompass views on safety, health, comfort, productivity, quality, and overall satisfaction with workplace facilities and offer valuable insights [2,12]. A facility perceived as safe and healthy not only reduces the risk of accidents and health issues but also fosters a sense of well-being among employees. Comfortable facilities contribute to employee satisfaction and can positively impact productivity. Furthermore, the perceived quality of workplace facilities reflects on the company's image and can influence employee morale. Ultimately, employee satisfaction with their workplace environment affects retention rates, job performance, and the organization's overall success [13,14]. Therefore, it can be confirmed that various things need to be considered in facility management. It covers a scope that involves the management of the environment, assets, and people.

These challenges underscore the importance of developing a comprehensive model to guide safety and health practices in building management. The need for such a model arises from the complex interplay between the physical environment, facility management, and worker perception. These elements collectively influence the overall quality of the workplace and, consequently, employee well-being and productivity. A well-structured model can provide organizations with a systematic approach to addressing these factors, leading to enhanced safety, health, and satisfaction among workers. Furthermore, such a model can serve as a benchmark for continuous improvement in facility management practices, ensuring that workplaces remain conducive to high performance and employee satisfaction. Hence, the objective of this current research is to develop a model for safety and health practices in building, focusing on the physical environment, facility management, and worker perception in the Federal Territory of Putrajaya. The aim is to assess and improve these practices to enhance the quality of the workplace for employees.

## 2 Literature review

Malaysia, as a rapidly developing nation striving to achieve developed status with a world-class infrastructure, has witnessed a surge in well-planned development initiatives. Notably, the construction sector, which plays a pivotal role in meeting societal and organizational demands, has seen substantial growth. However, amid the swift progress of building construction projects, particularly within the government buildings of the federal territory of Putrajaya, the critical aspect of facilities management planning should not be overlooked [15]. Nowadays, a multitude of concerns pertaining to quality, safety, and health have emerged, largely stemming from deficiencies in facilities management, particularly in building maintenance [11,16]. These issues encompass structural impairments, fire hazards, structural collapse risks, and legal non-compliance. These challenges underscore the need for a comprehensive model that addresses safety and health practices within office buildings.

Despite extensive research in various aspects of facility management, physical environment, and worker perceptions, a holistic model that integrates these dimensions comprehensively is conspicuously absent. Existing studies often focus on occupational safety and health aspects without addressing the interplay between facility management practices, the physical work environment, and the perceptions of office workers. For instance, a study conducted by Hauashdh et al. and Halim et al. were focused on facility management of building maintenance practices [11,17], Utomo et al. and Van Tran et al. were focused on physical environment of building [8,18], while De Simone and Fajilla focused on safety perception of workers toward their workplaces [19]. This fragmentation leads to gaps in effectively ensuring building office safety and health.

To develop a comprehensive safety and health practices model tailored to office buildings, this study identifies three main dimensions of relevance. The first dimension focuses on the implementation of facility management in occupational safety and health practices. This involves aspects such as objective setting, policies and strategies; implementation of planning; cost allocation; procedure development; compliance with regulations and standards; and workforce utilization [7,11,17]. Secondly, the study aims to determine key factors in the office's physical environment that could lead to unsafe and unhealthy conditions for workers, including indoor air quality; lighting; noise levels; cleanliness; finishing and structure of the building; and building services [6,8,18,20,21]. Thirdly, the researcher examines the perceptions and impacts faced by office workers through the contributions of facility management to aspects of safety, health, comfort, productivity, service quality, and satisfaction [19,22,23].

### 2.1 First dimension: facilities management

Facility management has been formed to describe an organization that connects strategic, operational and tactical aspects in providing services that prioritize the safety and health of employees in the office. Nutt explained that facility management includes issues related to the physical condition of the building space, services, technology, maintenance, modification, and adaptation; human and business concerns regarding facility purpose, function, and usage; security, safety, environmental health, and comfort; financial issues regarding real estate investment, asset value, cost, and profitability [24]. Moore and Finch and Alsayyari et al. also added that facility management is generally involved in several disciplines and services and often confusing usage in describing certain areas [25,26]. Previous studies have identified and evaluated many facility management processes such as customer requirements management, service management, supervision, contract management, health and safety management, risk management, and others [[27], [28], [29], [30], [31]]. In this study, the dimension of facility management is divided into six parameters as follows:

i. Objectives, Policies and Strategies

According to Koleoso et al. and Nawi et al., facility management organizations should strive to form objectives, policies, and strategies that emphasize aspects of maintaining a safe and healthy workplace environment for all parties, including facility management employees, building occupants, visitors and the neighbourhood [32,33]. Objectives should be clear, measurable, and aligned with the overall safety and health goals of the organization [34]. Policies must be comprehensive, covering all aspects of workplace safety, health regulations, and emergency procedures [35]. Strategies should include regular reviews and updates to ensure they remain relevant and effective in addressing emerging safety and health challenges. By prioritizing these elements, facility management can foster a safety and health culture, ensuring that all activities and decisions are guided by these principles [34,35].

ii. Planning

Effective planning is crucial in facility management, particularly in the safety and health aspects [[36], [37], [38], [39], [40]]. Planning should prioritize preventive measures over corrective actions to minimize risks and enhance safety. This involves short-term (up to 2 years), intermediate (2–5 years), and long-term (5–10 years) planning periods. Short-term planning focuses on routine maintenance and immediate safety measures, ensuring that all systems and equipment are functioning correctly and safely [41]. Intermediate planning addresses more substantial maintenance needs and upgrades, preparing for potential changes in regulations or the introduction of new technologies [42]. Long-term planning involves strategic investments in infrastructure, anticipating future safety and health requirements, and ensuring the sustainability of facility management practices [41,42]. Each planning stage should include detailed risk assessments, resource allocations, and performance metrics to monitor and evaluate the effectiveness of implemented measures.

iii. Financial

Financial planning in facility management is essential to balance the annual work schedule and planned activities [43]. Oseghale states that an appropriate budget must be prepared based on expected costs for facility management activities, ensuring that all necessary resources are available for maintaining safety and health standards [43]. This includes funding for regular maintenance, emergency repairs, staff training, and compliance with legal requirements. Financial allocations should be flexible to accommodate unforeseen expenses or changes in regulations. A robust financial plan enables facility management organizations to prioritize safety and health investments, ensuring that all activities are adequately funded and aligned with the organization's strategic objectives.

iv Procedure

Facility management procedures are critical technical documents that guide daily activities to ensure safety and health [44,45]. Zakaria and Arifin and Isa et al. emphasized that management should take precautions to ensure that employees and visitors are not exposed to any hazards due to the activities being carried out in the workplace [44,45]. Procedures should be comprehensive, covering all aspects of facility operations, including maintenance routines, emergency responses, and safety protocols. They should be regularly reviewed and updated to reflect changes in regulations, technology, and best practices [41]. Effective procedures facilitate consistent and safe execution of facility management activities, providing clear guidelines for staff and ensuring compliance with safety and health standards.

vi. Rules and Standards

Rules and standards are essential for determining whether an organization or individual meets the desired output or achievements of a facility management organization. Rules and standards consist of guidelines, measurements, and reference sources to determine whether an organization or individual should carry out relevant activities to meet the output or achievements of a facility management organization. Thus, the standards and standards used by the facility management organization are aimed at providing quality services for building maintenance for the comfort, safety, and health of all occupants [41]. Mariah and Mohammad support the idea that regulations and standards are also used as a reference source for employers and professionals in the field of safety and health, medical practitioners, and engineers [46]. Adherence to established rules and standards ensures that facility management practices are aligned with industry benchmarks, promoting a safe and healthy workplace environment. Regular audits and assessments are essential to verify compliance and identify opportunities for improvement.

vii. Human resources

Providing sufficient human resources or workforce to carry out tasks related to building maintenance is the full responsibility of the facility management organization. This includes both skilled and semi-skilled workers who ensure that building facilities are adequately maintained [10,47]. Skilled workers, with their expertise and experience, play a crucial role in performing complex maintenance tasks and leading teams. They also act as mentors for semi-skilled workers, providing training and guidance to enhance their capabilities. Effective human resource management involves continuous training and development programs, ensuring that all workers are knowledgeable about the latest safety and health standards and practices [41]. Adequate staffing levels are essential to meet the demands of facility maintenance, ensuring that all tasks are completed efficiently and safely.

### 2.2 Second dimension: physical environment

Safety and health in buildings are divided into three main components, namely physiological, psychological, and environmental. Gola et al. and Waring stated that the physical factors of the workplace environment directly affect occupational safety and health [20,48]. This recent research studies six parameters associated with the physical environment dimension. All the physical environment factors studied are related to the facilities in a building, and they need to be maintained accordingly to ensure the safety and health of all workers.

i. Indoor air quality

The indoor air of a building is often affected by the air conditioning system and air circulation system [8,23]. Good indoor air quality is needed by every employee who prioritizes a healthy indoor environment to improve their comfort. According to Kasnavi and Fathalizadeh, there are two most frequently reported complaints about indoor air quality, namely discomfort and illness [49]. A study by Ahmad and Hassim found that poor air quality is closely related to air conditioning system operation and maintenance problems [50]. There are four sub-parameters for indoor air quality, namely temperature, relative humidity, ventilation flow rate and odour.

ii Lighting

Lighting is vital for safety and health factors in the physical environment of workers at work [18,51,52]. Inappropriate lighting (i.e. dim or glare) will affect the health or safety of workers in a building. Employees will face eye discomfort, short-sightedness, or watery eyes caused by working in a workstation with poor lighting or glare [52]. In addition, insufficient lighting can also lead to accidents, especially for workers who carry out high-risk jobs such as mechanical and electrical system maintenance. Moreover, the risk of slips, trips and falls is also an effect of insufficient lighting.

iii. Noise levels

Two main factors often cause noise problems in the building office, the first being the operation of building service systems such as chiller-type air conditioning systems, water pump systems, and standby power [21]. Secondly, caused by office cleaning equipment such as vacuums and carpet cleaners. The level of discomfort for adults to sound is between 85 and 95 dB A, and the pain level is between 120 and 140 dB A [53]. The maximum sound intensity that can be received by the human ear continuously without hearing damage is in the range of 90 dB A to 100 dB A [54].

iv Cleanliness

The aspect of cleanliness is often taken for granted and not given serious attention, causing the level of cleanliness of an area or part of a building to be very disappointing [55,56]. Good hygiene practices play an essential role in occupational health as well as ensuring a safe workplace. Accidents will easily occur if cleanliness in the office is neglected, such as fire, slipping, tripping, falling and so on, resulting in fatality, injury, and property damage [57]. Besides that, health disorders are also easily caused by a low level of hygiene through the reproduction of bacteria, pest attacks, and fungal growth in the office [40].

v. Finishing and structure of the building

The assumption that a building is a place that is always safe and protected from various safety and health threats is not valid. Collapsing ceilings, leaking roofs or windows, cracked walls or floors, peeling paint, broken glass doors or windows, damp walls, and others are office defects that office workers will face. All defects stated can contribute to office workers' fatalities, injuries, or illnesses. Olubodun and Mole and Albert et al. explained that five main factors cause building defects: office design factors, the standard of materials used, the quality of construction, the age of the office and vandalism [58,59]. Yacob et al. added that three of the five building defect factors that have been explained (i.e. office design factors, the standard of materials used, and the quality of construction) are caused during a stage of building in the process of planning and construction and the other two factors (i.e. the age of the office and vandalism) after the office is in operation [60].

vi. Building services

Building services are facilities equipped in offices and used directly by building workers, such as passenger lifts, escalators, electricity supply, water supply, and air conditioning [61,62]. Workers who use building services are also exposed to various safety and health risks. The level of risk for each building service depends on how the facility management manages it [63].

### 2.3 Third dimension: perceptions of office workers

It is crucial to ensure the environment in the office is at a comfortable, safe and healthy level because the duration of time employees in the office is long, which is approximately 8–9 h a day and involves 5–6 days a week. Therefore, it is clear that efficient facility management reflects a contribution that significantly impacts providing high-performance workplace facilities [64,65]. Perception and positive impact on office workers is an essential agenda of a facility management organization. Office workers should always be informed about any issues or problems to prepare workers mentally and physically for possible negative consequences. The office environment can affect how people work and their achievements, and it also can contribute to human errors in doing work and experiencing work stress. According to a study conducted by Clement-Croome, employee productivity can be increased by 4 %–10 % by improving the office environment [66]. This study explores the dimension of office worker's perceptions is through six parameters as follows:

i. Safety

All employees want a safe workplace while in the office [59,67,68]. Any accident that occurs can have an impact, such as trauma, injury, disability, or fatality [69]. Ensuring a safe workplace involves implementing comprehensive safety protocols, conducting regular safety drills, and maintaining clear communication channels for reporting and addressing safety concerns. The presence of adequate safety measures, such as fire alarms, emergency exits, and first-aid kits, reassures employees and fosters a secure working environment.

ii Healthy

The health of employees should remain consistent before, during, and after office hours if the office environment is conducive to well-being [70]. Poor indoor air quality, inadequate ventilation, and other environmental factors can contribute to building-related illnesses or sick building syndrome, manifesting as headaches, nasal congestion, chest congestion, dry skin, fatigue, and more [71]. Regular monitoring and maintenance of HVAC systems, ensuring access to natural light, and promoting ergonomic workspaces are essential strategies to support employee health.

iii. Comfortable

Employees will be at more than one-third per day and at least 5 days a week at a workplace. The office, often considered a "second home" by employees, requires a comfortable environment to foster productivity and job satisfaction [72]. Facility management should ensure ergonomic furniture, appropriate lighting, optimal temperature control, and minimal noise levels. Comfortable office environments reduce physical strain and mental stress, enabling employees to focus better and perform their tasks efficiently. Additionally, providing amenities such as break rooms, rest areas, and recreational spaces can enhance overall comfort [72].

iv Productivity

Every employer expects his employees to contribute high productivity to their work. According to research, high work productivity can only be achieved if the workplace environment is satisfactory [73,74]. A well-designed office space can significantly boost productivity by minimizing distractions and providing necessary resources for efficient work. This includes access to advanced technology, collaborative spaces, and quiet zones for focused work. Moreover, a supportive workplace culture that values and recognizes employee contributions further enhances productivity.

v. Quality

The quality of work produced by an employee is closely related to the workplace environment [16,75]. Employees are able to focus on the given work and produce good quality work if they feel comfortable with the workplace provided by the facility management organization. Factors such as cleanliness, organization, and the availability of quality resources play a crucial role in enabling employees to concentrate and produce superior work outcomes. Facility management should prioritize continuous improvement of office conditions to sustain high work quality.

vi. Satisfaction

Employee satisfaction with their workplace environment can influence their willingness to stay with the organization. Satisfaction is associated with the relief, enjoyment and pleasure that employees feel towards the workplace provided [76]. A positive and supportive office environment reduces turnover rates and enhances employee loyalty. Regular feedback mechanisms, employee engagement programs, and addressing workplace grievances promptly contribute to higher levels of satisfaction. Ensuring that the office environment aligns with the needs and preferences of employees fosters a sense of belonging and commitment to the organization.s.

## 3 Research methodology

### 3.1 Population and sample of the study

This study was conducted within the office complexes around the Federal Government Administrative Center in the Federal Territory of Putrajaya, Malaysia. The selection of this research area is based on complaints often heard about public government buildings having a low level of maintenance, safety and cleanliness [15] compared to private buildings which are usually better maintained [77]. Halmetoja and Lepkova, in their study, said that employees who work in private organizations find it relatively easy to control the work environment, feel high environmental satisfaction and feel favourable ambient conditions compared to those working in the government sector who feel less satisfaction and lack control over the office environment [78]. A total of nine government office complexes were chosen as this study location.

Regarding the selected study location, the study population and sample is a group of government office workers consisting of three groups, namely: (1) top management officers, (2) management and professional officers, and (3) supporting staff. This group is a government office worker that always used and exposed to physical conditions in an office. Therefore, the data and information from the group are very relevant to this current study.

The total population of government office workers for all nine office blocks is 5,618, where 81 are categorized as top management officers; 1987 as management and professional officers; and 3559 are categorized as supporting staff. This study includes a sample of 562 respondents, categorized as follows: 8 top management officers, 198 management and professional officers, and 356 supporting staff members. The total population and sample of government office workers in this study are summarized in Table 1. The sample size aligns with recommendations by Iacobucci [79] and Kline [80], with Iacobucci [79] suggesting that 150 respondents are adequate for SEM analysis, while Kline [80] notes that samples below 100 are generally unsuitable for such analyses.

**Table 1:** Total population and sample of government office workers.

Study location	Total Population			Total Sample		
	Top management officers	Management and professional officers	Supporting staff	Top management officers	Management and professional officers	Supporting staff
Complex A	11	252	181	1	25	18
Complex B	8	136	467	0	14	47
Complex C	7	174	491	1	17	49
Complex D	10	291	341	0	29	34
Complex E	9	198	231	1	20	23
Complex F	10	269	505	1	27	51
Complex G	10	287	472	0	29	47
Complex H	8	97	361	1	10	36
Complex I	8	274	510	3	27	51
Total	81	1978	3559	8	198	356

To minimize social desirability bias, this study followed confidentiality and anonymity measures, as suggested by Nederhof [81] and Krumpal [82]. The survey consisted of a cover letter and a questionnaire, with the cover letter emphasizing that participation was voluntary and the guarantee of response confidentiality. The questionnaire intentionally omitted personal information such as names and signatures to preserve respondent anonymity. Additionally, the questionnaire featured a combination of positively and negatively worded questions and utilized specific wording to minimize social desirability bias, foll.owing the approach outlined by Krumpal [82].

### 3.2 Data collection

Data collection is through a survey form that contains 4 main sections. Section A is about the respondent's background, namely gender, age, duration of time working in the office, and duration of time a day in the office. Section B is an assessment of facility management dimensions in safety and health practices which comprised six parameters, namely objectives, policies, and strategies; planning; financial; procedures; rules and standards; and human resources. Section C, in contrast, assesses the dimensions of the physical environment factors of safety and health based on 6 main parameters, namely indoor air quality; lighting; noise; cleanliness; finishing and structures of building; and building services. Lastly, Section D determines office workers' perception of facility management and its impact on safety and health throughout the month before the survey is conducted. This section is divided into six parameters related to office workers' perception of office physicality based on the components of safety, healthy, comfort, productivity, quality, and satisfaction.

The research data were analyzed using the Statistical Package for the Social Sciences (SPSS) software and AMOS version 23.0 to obtain the study results. SPSS is a handy software for data management and analysis, especially in social science research [83,84]. On the other hand, AMOS software was used to analyze data for Structural Equation Modeling (SEM) analysis to achieve the research objective, which is to develop a safety and health practices in building model of physical environment, facility management, and worker perception.

### 3.3 Model development

**Fig. 1:**
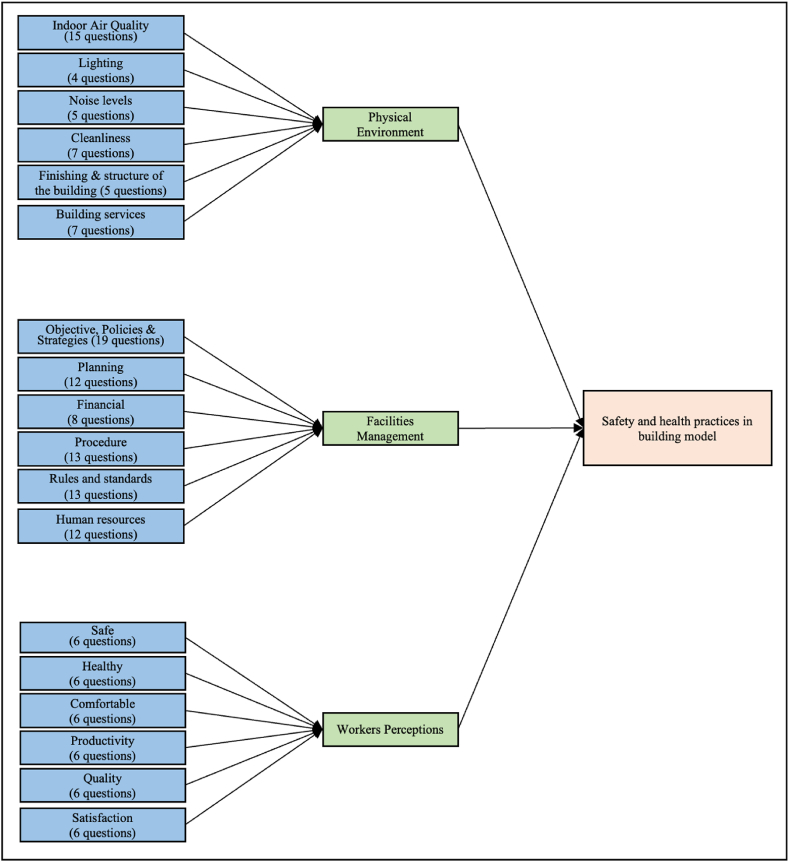
A model framework for a safety and health practices in building.

Drawing from the literature review, a model framework for a safety and health practices in building has been constructed, as illustrated in Fig. 1, to explore the relationship among latent and observed variables. This framework is structured as a measurement model, often referred to as a hypothetical model, which illustrates connections between latent variables, observed indicators, and variance errors [85,86]. Referring to Fig. 1, latent variables consist of all dimensions of the safety and health practices in building model: facilities management, physical environment, and perceptions of office workers. The observed variables are represented by 156 items (questions) for all three dimensions under the safety and health practices in building model. Data for all observed variables were gathered from respondents located in selected government office buildings.

Before the SEM test is performed, the normality of the data distribution for each of the eighteen observed variables in the framework for a facility management index for safety and health practices development model should be tested. This determines whether the data meet the normality assumption of the Maximum Likelihood Estimation (MLE) method. The normality tests performed for each variable are skewness and kurtosis. This is because the use of SEM is very sensitive to the data distribution characteristics, and the MLE used in SEM is based on the assumption of multivariate normality [87]. The results of the normality test indicate that the data distribution is normal, as the skewness falls within the range of −2 to +2, and the kurtosis is between −10 and + 10 [83,85].

SEM is an analysis used to investigate cause-and-effect relationships between a group of variables in a study using the model testing method [85,86]. The model development in this research also combines path analysis and Confirmatory Factor Analysis (CFA). Path analysis is a way to represent a set of regression equations by using a causal diagram [88]. Path analysis also uses simple bivariate correlations to estimate relationships in a structural equation system [87]. In contrast, the CFA aims to measure the suitability of the model formed with the study data for all latent variables. If the measurement items (study data) do not fit the latent variable, then the research model development is unreliable [85].

## 4 Research results and discussions

### 4.1 Respondents' socio-demography

Table 2 illustrates in detail the socio-demographic characteristics of the respondents from the data collection. This research involved a total of 562 respondents, all of whom were government employees based in federal office buildings in Putrajaya. The majority of the respondents, i.e. 358 workers (63.7 %), were female, while the rest, i.e. only 204 workers (36.3 %), were male. The largest age group represented was 21–30 years old, comprising 60.1 % of respondents. This was followed by 20.3 % aged between 31 and 40, 10.0 % over 51, 9.1 % between 41 and 50, and only 0.5 % between 18 and 20 years old.

**Table 2:** Respondent's socio-demography.

Characteristics	Category	Frequency (f) (N = 562)		
		Top management officers	Management and professional officers	Supporting staff
Gender	Male (36.3 %)	8	78	118
	Female (63.7 %)	0	120	238
Age	18–20 years (0.5 %)	0	0	3
	21–30 years (60.1 %)	0	115	223
	31–40 years (20.3 %)	0	50	64
	41–50 years (9.1 %)	0	18	33
	51 years or more (10 %)	8	15	33
Duration of time working in the office	5 months or less (11.6 %)	1	28	36
	6–11 months (12.6 %)	0	30	41
	1–2 years (32.2 %)	2	68	111
	3–4 years (20.6 %)	3	41	72
	5–6 years (11.8 %)	0	18	48
	7 years or more (11.2 %)	2	13	48
Duration of time a day in the office	8 ½ hours or less (0.9 %)	0	1	4
	9 h (2.1 %)	0	4	8
	9 ½ hours or more (97.0 %)	8	193	344

In terms of job tenure, 32.2 % (181 workers) had worked in the study office for 1–2 years. This was followed by 20.6 % (116 workers) with 3–4 years of experience, and 12.6 % (71 workers) with 6–11 months of service. Additionally, 11.8 % (66 workers) had been employed for 5–6 years, 11.6 % (65 workers) had worked for 5 months or less, and 11.2 % (63 workers) had the longest tenure, having worked for 7 years or more.

The study also found that most government office workers were in the office for 9 and a half hours or more every day, reaching 97.0 % (545 workers) of respondents. In addition, 2.1 % (12 workers) were in the office for 9 h, and only 0.9 % (5 workers) were in the office for a period of less than 8 and a half hours. Working hours for every government office worker are subject to the Public Service Department through Service Circular Number 5 Year 2019 for the implementation of Flexible Working Hours (FWH) in all Federal Government organizations throughout the country. FWH is a flexible working time where employees are given the flexibility to attend work during the set entry and exit times, provided they meet the daily working hours. Government office workers' daily working time (not including breaks) is 8 h. This study's findings show that most government office workers spend over one-third of their daily time at work. This finding is in line with a previous study conducted by Huizenga et al. [72].

### 4.2 Normality test

The results of the normality test for all observed variables in the model framework for a safety and health practices in building are presented in Table 3. The test results found that all variables can be considered normal with skewness values in the range of - 1.060 to - 0.107 and kurtosis values - 0.947 to +4.244, which meet the guidelines stated by Mackey and Gass [83,85]. Therefore, the SEM test can be performed using the framework of the proposed development model and can produce good results.

**Table 3:** The result of the normality test.

Description	Skewness	Kurtosis
Facilities Management		
Objective, Policies and Strategies	−0.445	0.456
Planning	−0.811	2.454
Financial	−0.879	2.219
Procedure	−0.831	3.612
Rules and standards	−0.544	1.586
Human resources	−0.596	1.905
**Physical Environment**		
Indoor Air Quality	−0.089	−0.815
Lighting	−0.254	−0.947
Noise levels	−0.455	−0.488
Cleanliness	−0.107	−0.589
Finishing and structure of the building	−0.915	−0.209
Building services	−0.473	−0.787
**Perceptions of Office Workers**		
Safe	−1.060	3.557
Healthy	−0.757	2.743
Comfortable	−0.599	1.938
Productivity	−0.884	4.244
Quality	−0.448	4.001
Satisfaction	−0.324	1.668

### 4.3 Path analysis model

**Fig. 2:**
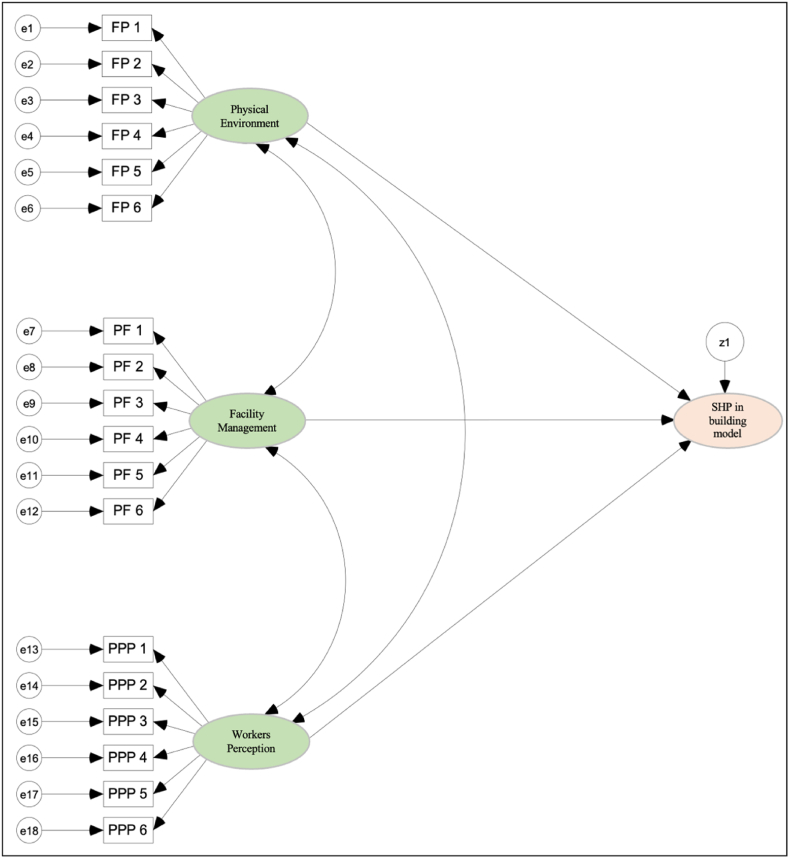
Path analysis Model.

A path analysis model is formed based on dependent (latent) and independent (observed) variables that influence safety and health practices in building. The dependent variables in this study include facility management, the physical environment and perception of office workers. In comparison, the independent variable is 156 measurement items forming the model. Fig. 2 shows the path analysis research model on a set of relationships that includes the variables in the study represented by a graphical representation using AMOS software. Parameters estimation for each relationship is done with the Maximum Likelihood Estimation (MLE) procedure which is an iterative technique to find the best solution for the path coefficient by identifying the minimum value for the fit function.

### 4.4 Evaluating path analysis model fit using CFA

After estimating the path analysis model of safety and health practices in building model development, its suitability should be evaluated using the Goodness of Fit measurements. Sarstedt [87] suggest three types of Goodness of Fit measurements that can be used, namely, Absolute Fit Index (AFI), Incremental Fit Index (IFI), and Parsimonious Fit Index (PFI). In the Absolute Fit Index measurement, three measurements are often used, the Chi-square analysis (Chi-square, χ^2^); Goodness of Fit Index (GFI); and the Root Mean Square Error of Approximation (RMSEA). The Chi-square test is used to assess the null hypothesis that 'the measurement model in the research model fitted with the study data'. So, if the Chi-square test is not significant (p-value> 0.05), this concludes that the model is significantly fitted. Meanwhile, a model is considered fit if the GFI value exceeds 0.90 and the RMSEA values are less than 0.10 [87,[89], [90], [91]].

For the second measurement, which is the Incremental Fit Index (IFI), three indices are often used to assess the fit of the model, namely the Adjusted Goodness-of-Fit Index (AGFI), the Tucker-Lewis Index (Tucker-Lewis Index, TLI) and Normed Fit Index (NFI). Index values for AGFI, TLI, and NFI that exceed 0.90 indicate that a model is fit.

The last measure in assessing model fit is the Parsimonious Fit Index which consists of three main measures, namely Parsimonious Normed Fit Index (PNFI), Parsimonious Goodness-of-Fit Index (PGFI), and Chi-Square Norm (Normed Chi-square, χ^2^/df). To assess the fitted measurement model, Blunch Blunch (2017) stated that the higher the PNFI and PGFI values obtained, the better the model. In the meantime, Kim et al. [92] stated that a model is considered fit if the χ^2^/df value is less than 3.

The Goodness of Fit measurements obtained for the path analysis model in this study are summarized in Table 4. Referring to Table 4, the result of the Chi-Square Goodness of Fit test is significant [χ^2^ (N = 562, df = 132) = 623.989, p < 0.05]. However, the path analysis model developed significantly does not match the data collected from the study respondents. This is because the GFI, RMSEA, AGFI, TLI, NFI, and *χ*^*2*^*/df* fit index values does not meet the evaluation criteria of the measurements.

**Table 4:** Goodness of Fit measurements of the path analysis model before modification.

Measurement	Index	Evaluation Criteria	Value
Absolute Fit Index, AFI	*χ*^*2*^		623.989
	*df*		132
	*p*	<0.05	0.000
	GFI	>0.90	0.889
	RMSEA	<0.10	0.082
Incremental Fit Index, IFI	AGFI	>0.90	0.856
	TLI	>0.90	0.900
	NFI	>0.90	0.894
Parsimonious Fit Index, PFI	PNFI	>0.50	0.771
	PGFI	>0.50	0.789
	*χ*^*2*^*/df*	<3.00	4.727

#### 4.4.1 Modification index

The results of the Goodness of Fit measurements (Table 4), which are significant suggest that the path analysis model that was formed does not match the research data. To achieve a satisfactory model fit, adjustments to the modification index are necessary to fulfill all fit index criteria, enabling the model to be significantly fitted with the research data [92]. Table 5 presents the values of the modification index to improve the fit of the model with the research data.

**Table 5:** Modification index (M.I).

Relationship			M.I	Par Change
e14	↔	e13	79.569	3.473
e17	↔	e13	35.484	−1.844
e17	↔	e14	28.610	−1.333
e17	↔	e16	81.404	1.809
e18	↔	Facility Management	20.696	2.637
e18	↔	e16	28.681	−1.220
e8	↔	e7	20.179	7.420
e12	↔	e11	22.747	3.305
e2	↔	e1	45.514	4.531
e6	↔	e5	43.798	2.600

**Fig. 3:**
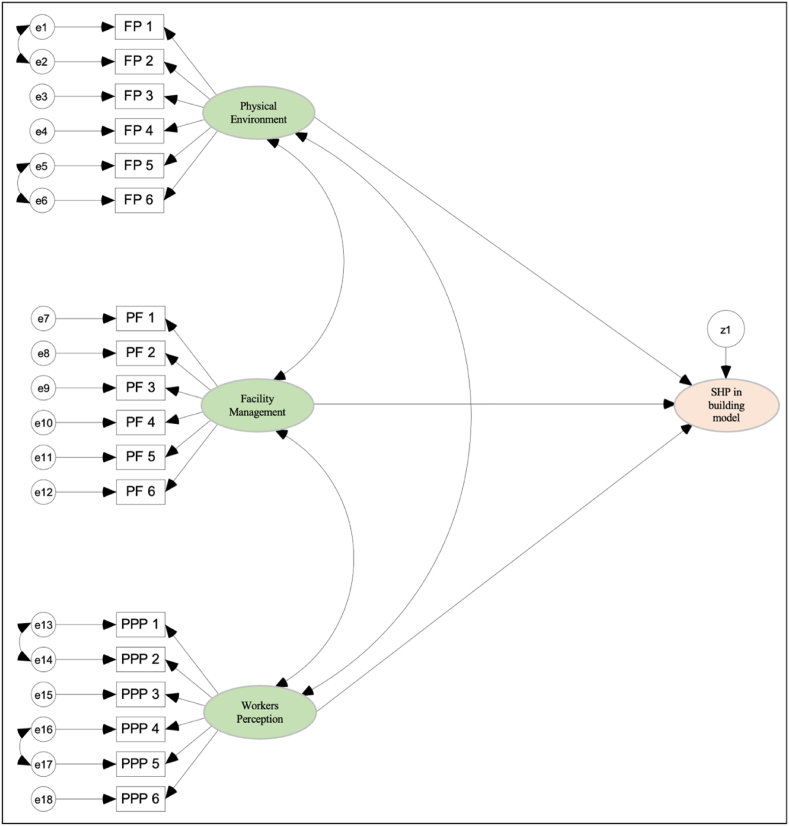
Modification model.

To improve the fit of the path analysis model with the research data, the correlation between several variables indicated by the modification index can be determined. Correlation between variables with a high modification indicator value will reduce the probability of the significance of the Chi-Square value and directly increase the model's fit with the research data. However, Chua [85] highlighted that every correlation of variables to be done needs to consider theoretically whether there is a relationship between the variables. If the literature shows a relationship between the two variables, then the two variables can be correlated in the model. Based on the modification index and previous studies, four pairs of variables are proposed to be correlated, namely e17 (quality) ↔ e16 (productivity); e14 (healthy) ↔ e13 (safe); e2 (lighting) ↔ e1 (indoor air quality); and e6 (building services) ↔ e5 (finishing and structure of the building). Fig. 3 shows the path analysis model after modifications were made for the development of the facility management index for safety and health practices.

Table 6 shows the Goodness of Fit measurements obtained for the path analysis model after the modifications were carried out. The results of the Chi-Square Goodness of Fit test showed a change after the base model was modified but were still significant [χ^2^ (N = 562, df = 128) = 376.836, p < 0.05]. As shown in Table 6, the modification process satisfied all fit index criteria. The fit indices, including GFI, RMSEA, AGFI, TLI, NFI, and χ^2^/df, all met their evaluation thresholds when compared to the base model in Table 5. This indicates that, after modification, the model aligns significantly better with the study data than the original base model.

**Table 6:** Goodness of Fit measurement of the path analysis model after modification.

Measurement	Index	Evaluation Criteria	Value
Absolute Fit Index, AFI	*χ*^*2*^		376.836
	*df*		128
	*p*	<0.05	0.000
	GFI	>0.90	0.932
	RMSEA	<0.10	0.059
Incremental Fit Index, IFI	AGFI	>0.90	0.909
	TLI	>0.90	0.948
	NFI	>0.90	0.936
Parsimonious Fit Index, PFI	PNFI	>0.50	0.783
	PGFI	>0.50	0.800
	*χ*^*2*^*/df*	<3.00	2.944

#### 4.4.2 Variable regression

The next test is a regression against each dependent variable, namely facility management, physical environment, and perception of office workers, with independent variables. The regression test produces the path coefficient value, which has been estimated using the standardized regression coefficient (β weights) value for each relationship between the dependent and independent variables (Table 7). Relationship estimation is done using the Maximum Likelihood Estimation (MLE) procedure. The path coefficient values reflect the relative strength of the variables that develop the facility management index model in safety and health practices. It is also used to determine a dominant variable that affects a dependent variable.

**Table 7:** Standard regression coefficient value (β) against the path analysis Model.

Dimension	Path			β value	p-value
Physical Environment	Physical Environment	→	Indoor Air Quality	0.750	0.00∗
	Physical Environment	→	Lighting	0.668	0.00∗
	Physical Environment	→	Noise levels	0.716	0.00∗
	Physical Environment	→	Cleanliness	0.840	0.00∗
	Physical Environment	→	Finishing and structure of the building	0.700	0.00∗
	Physical Environment	→	Building services	0.778	0.00∗
Facilities Management	Facilities Management	→	Objectives, Policies and Strategies	0.753	0.00∗
	Facilities Management	→	Planning	0.803	0.00∗
	Facilities Management	→	Financial	0.718	0.00∗
	Facilities Management	→	Procedure	0.869	0.00∗
	Facilities Management	→	Rules and standards	0.882	0.00∗
	Facilities Management	→	Human resources	0.844	0.00∗
Perceptions of Office Workers	Perceptions of Office Workers	→	Safe	0.559	0.00∗
	Perceptions of Office Workers	→	Healthy	0.701	0.00∗
	Perceptions of Office Workers	→	Comfortable	0.723	0.00∗
	Perceptions of Office Workers	→	Productivity	0.702	0.00∗
	Perceptions of Office Workers	→	Quality	0.647	0.00∗
	Perceptions of Office Workers	→	Satisfaction	0.721	0.00∗
Indexes	Physical Environment	→	Indexes	0.432	0.00∗
	Facilities Management	→	Indexes	0.649	0.00∗
	Perceptions of Office Workers	→	Indexes	0.259	0.00∗

∗ Significant at the p < 0.01 level.

Table 7 clearly shows the direct effect of the independent variables on the dependent variables that develop the model based on the significance of the β coefficient. The test results also show that the value of the standardized regression coefficient is between 0.259 and 0.882. This indicates that all independent variables can significantly represent each dependent variable.

The results of the analysis found that all three factors, namely facilities management, the physical environment, and perceptions of office workers are significant, with the most dominant factor being facility management (0.649), followed by the physical environment factor (0.432), and perceptions of office workers (0.259). This finding is in line with previous studies by Jaafar et al. [63]; Utomo et al. [18]; and Van Tran et al. [8]. Moreover, the results also describe that the elements of objectives, policies, and strategies; planning; financial; procedures; rules and standards; and human resources significantly influence respondents on the dimension of facilities management. Based on the regression coefficient values, it can be concluded that the elements of rules and standards are more dominant (0.882) compared to other elements that influence respondents on the dimension of facilities management. It is undeniable that rules and standards pertaining to facilities management are essential for minimizing the risk of workplace accidents and illness as stressed by Reuter et al. [10]. Therefore, safety and health rules must be established in every facility management organization to meet local, national, and international legislation.

As for the dimension of the physical environment, the indoor air quality parameters; lighting; noise level; cleanliness; finishing and structure of the building; and building service significantly affected respondents on this dimension. The cleanliness parameter (0.840) was found to be more dominant in influencing respondents on the physical environment dimension than other elements. Finally, the findings of this study discover that the parameters of safe; healthy; comfortable; productivity; quality; and satisfaction significantly affected respondents' dimensions of perception of office workers. Based on the regression coefficient values, it can be concluded that the comfortable element (0.723) is more dominant than the other elements that influence respondents on the dimension of perception of office workers. Hence, the comfort element which is closely related to work performance and productivity needs to be emphasized as stated in previous research by Mallawaarachchi and De Silva [23] and Animashaun and Odeku [64].

#### 4.4.3 Variable correlation

The findings in Table 8 show a significant positive linear correlation at the p < 0.01 level against the path analysis model for the development of the facility management index for safety and health practices. Based on the value of the correlation coefficient, the relationship that exists is weak which is r = 0.310–0.500 between facility management parameters and the perception of office workers (r = 0.448), which suggest that better facility management leads to improved worker perceptions of safety. On the other hand, e16 (productivity) with e17 (quality) (r = 0.427), e13 (safe) with e14 (healthy) (r = 0.369), and e5 (finishing and structure of the building) with e6 (building services) (r = 0.319). Are all positively correlated, indicating that enhancing one can improve the other. In addition, the results of this study also found that there is a very weak relationship which is r = 0.010–0.300 between the physical environment parameters with the perception of office workers (r = 0.282), the facility management with physical environment (0.175), and e1 (safe) with e2 (healthy) (r = 0.302). Although some relationships between variables are weak, they provide insights into interconnected factors influencing safety and health practices in office building.

**Table 8:** Value of correlation (r) against path analysis.

Correlation	*R* value	p value
Facility management ↔ Physical Environment	0.175	0.00∗
Facility management ↔ Perceptions of Office Workers	0.448	0.00∗
Physical environment ↔ Perceptions of Office Workers	0.282	0.00∗
e1 ↔ e2	0.302	0.00∗
e5 ↔ e6	0.319	0.00∗
e13 ↔ e14	0.369	0.00∗
e16 ↔ e17	0.427	0.00∗

∗ Significant at the p < 0.01 level.

### 4.5 Index development model

**Fig. 4:**
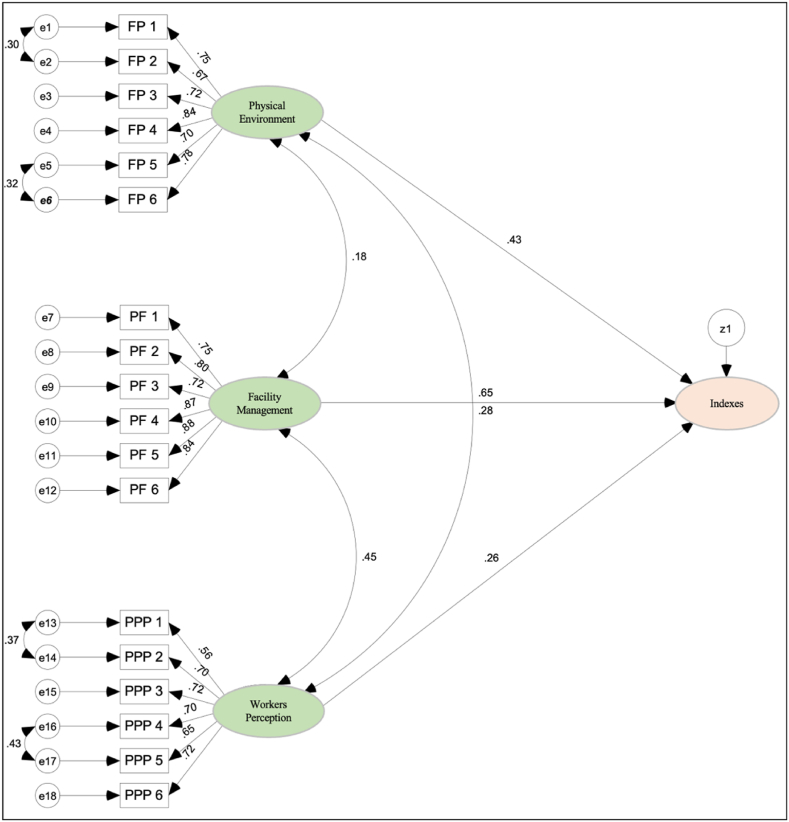
Safety and health practices in building model of physical environment, facility management, and worker perception.

Fig. 4 illustrates the overall development model of safety and health practices in building. This model can be considered as a fitted formation model for this study. This is because the developed model complies with the conditions of fit through the goodness of fit measurements of the model that have been carried out against each dependent variable and independent variable as suggested by Arbuckle [89]; Blunch [90]; Chen and Fong [91]; and Sarstedt [87]. The dependent variables described in this model consist of the dimensions of facility management, physical environment, perception of office workers and indexes. In comparison, the independent variable consists of 18 parameters.

This model also shows the values of the path coefficient, which is the standard regression coefficient (β) value, to explain the strength of the relationship between the variables that make up the safety and health practices in building model. Besides that, the correlation (r) that exists between the variables is shown in the model as in Fig. 4.

## 5 Conclusions

Occupational safety and health issues are important especially for the facilities management in order to ensure the safety, health and welfare of the employees. There are various issues related to safety and health associated with office workers, for instance, facility damage, building cracks, roof leaks, ventilation system issues, and other problems. These problems are closely related to the importance of facilities management. This current study focused on the three dimensions covering facility management, the physical environment, and the perception of office workers by using a quantitative analysis approach.

The findings reveal that facility management is the most significant factor influencing safety and health practices, followed by the physical environment and worker perceptions. The dominance of facility management (path coefficient of 0.649) suggests that adherence to rules and standards (β = 0.880) is crucial in mitigating occupational risks. Cleanliness emerged as the most critical element within the physical environment (β = 0.840), highlighting the importance of maintaining a clean workplace to ensure safety and health. The perception of office workers, particularly their comfort and satisfaction (β = 0.723), also plays a significant role, linking directly to their productivity and overall well-being.

The CFA test result validates that all three dimensions and their respective aspects have acceptable fit indexes (goodness of fit). Finally, the safety and health practice in building model of physical environment, facility management, and worker perception has been successfully developed to achieve the aim of this current study. The model offers a comprehensive understanding and facilitates the facility management organization's ability to effectively and systematically assess and monitor workplace conditions to provide a safe and healthy workplace environment. Hopefully, using and guiding this model by the stakeholders can prevent occupational safety and health issues from occurring in the future among office workers in the buidings.

A limitation of this study is that it focuses solely on the government offices around the federal territory of Putrajaya, Malaysia, which is focused on administration work. For future studies, authors are encouraged researchers could cover buildings owned by private companies to determine the differentiation with the facilities management by the government offices. Moreover, future studies would need to be conducted in other countries with various types nature of business (other than administration work) so that the facility management index model developed may be generalizable to a broader context within building environments. Furthermore, social desirability bias could influence respondents' answers to the questionnaires. However, to mitigate this bias, participants were assured of both confidentiality and anonymity throughout the research process.

## CRediT authorship contribution statement

**Mohammad Lui Juhari:** Writing – original draft, Visualization, Validation, Methodology, Formal analysis, Data curation. **Kadir Arifin:** Writing – original draft, Visualization, Validation, Supervision, Resources, Project administration, Methodology, Funding acquisition, Conceptualization. **Kadaruddin Aiyub:** Writing – original draft, Validation, Supervision, Methodology, Investigation, Conceptualization. **Zitty Sarah Ismail:** Validation, Project administration, Data curation.

## Data availability statement

Data associated with this study has not been deposited into a publicly available repository because the authors do not have permission to share data.

## Declaration of competing interest

The authors declare that they have no known competing financial interests or personal relationships that could have appeared to influence the work reported in this paper.
